# Effects of GH and IGF1 on Basal and FSH-Modulated Porcine Sertoli Cells In-Vitro

**DOI:** 10.3390/jcm8060811

**Published:** 2019-06-06

**Authors:** Rossella Cannarella, Francesca Mancuso, Rosita A. Condorelli, Iva Arato, Laura M. Mongioì, Filippo Giacone, Cinzia Lilli, Catia Bellucci, Sandro La Vignera, Riccardo Calafiore, Giovanni Luca, Aldo E. Calogero

**Affiliations:** 1 Department of Clinical and Experimental Medicine, University of Catania, 95123 Catania, Italy; rosita.condorelli@unict.it (R.A.C.); lauramongioi@hotmail.it (L.M.M.); filippogiacone@yahoo.it (F.G.); sandrolavignera@unict.it (S.L.V.); acaloger@unict.it (A.E.C.); 2 Department of Experimental Medicine, University of Perugia, 06132 Perugia, Italy; francesca.mancuso@unipg.it (F.M.); iva.arato@libero.it (I.A.); cinzia.lilli@unipg.it (C.L.); catia.bellucci@unipg.it (C.B.); Giovanni.luca@unipg.it (G.L.); 3 Department of Medicine, University of Perugia, 06132 Perugia, Italy; riccardo.calafiore@unipg.it

**Keywords:** FSH, IGF1, GH, Sertoli cells, AMH

## Abstract

Several lines of evidence suggest that insulin-like growth factor 1 (IGF1) is involved in Sertoli cell (SC) proliferation and that its receptor (IGF1R) could mediate follicle-stimulating hormone (FSH) effects. To examine the role of the growth hormone (GH)-IGF1 axis on SC function, we evaluated the effects of GH and IGF1 on basal and FSH-modulated SC proliferation, as well as on anti-Müllerian hormone (AMH) and inhibin B expression and secretion in-vitro. SCs from neonatal pigs were incubated with (1) placebo, (2) 100 nM highly purified urofollitropin (hpFSH), (3) 100 nM recombinant GH (rGH), (4) 100 nM recombinant IGF1 (rIGF1), (5) 100 nM hpFSH plus 100 nM rGH, (6) 100 nM hpFSH plus 100 nM rIGF1, for 48 h. We found that IGF1, but not FSH nor GH, stimulated SC proliferation. Furthermore, an inhibitory effect of FSH, GH and IGF1 on AMH secretion, and a stimulatory role of FSH and IGF1, but not GH, on inhibin B secretion were found. These results suggest that the GH-IGF1 axis influences basal and FSH-modulated SC proliferation and function. We speculate that SC proliferation occurring in childhood might be supported by the increased serum IGF1 levels observed during this period of life.

## 1. Introduction

Sertoli cells (SCs), which represent the major component of the testicular volume in children [1], are mainly committed to sustain spermatogenesis in adulthood. Therefore, an appropriate number of SCs is crucial for male fertility due to structural support, the role they play in the blood–testis barrier and the nourishment that they supply for germ cells (GCs) [2,3]. 

The insulin/IGF (insulin-like growth factor) signaling pathway has been suggested as one of the major hormonal signals involved in the establishment of a normal-sized cohort of SCs [4]. Pitetti and colleagues provided evidence supporting the importance of both insulin and IGF1 receptors (INSR and IGFR, respectively) in the regulation of SC proliferation, adult testis size and sperm output. Indeed, the weight of the testis of adult mice lacking the *Insr* (SC-*Insr*), the *Igf1r* (SC-*Igf1r*) or both (SC-*Insr;Igf1r*) in SCs is lower compared to wild type ones (weight decrease: 13.6%, 34.6% and 72.4%, respectively) and SCs have a lower proliferation rate during fetal and early neonatal life. This suggests that both insulin and IGF1 are required for the proliferation of immature SCs. Testis from SC-*Igf1r* and SC-*Insr;Igf1r* mice also showed a 38.85% and a 58.71% decrease in epididymal sperm concentration, respectively. Furthermore, in-vivo experiments indicated a role for insulin/IGF signaling in mediating the follicle-stimulating hormone (FSH) effect. Accordingly, FSH administration failed to increase SC number and testis weight in SC-*Insr;Igf1r* mice compared to control animals. Finally, inhibin B gene expression decreased compared to wild type, suggesting that insulin/IGF signaling is needed for FSH-stimulated inhibin B expression. On the contrary, anti-Müllerian hormone (*Amh*) gene expression was not affected [4]. 

AMH and inhibin B are dimeric glycoproteins secreted by SCs, both belonging to the transforming growth factor-β superfamily. The AMH serum levels reflect the number of immature SCs. Therefore, before puberty, because of the SC immaturity, AMH levels are high, thus representing a possible marker of SC function. At puberty, SCs mature and enter in a quiescence state, therefore, consequently, AMH production decreases and its serum levels decline. The rise in FSH levels, consistent with the beginning of puberty, cause an increase of inhibin B serum levels [1].

The growth hormone (GH) is released by the pituitary gland in a pulsatile manner and it is regulated by the hypothalamic hormones GH-releasing hormone (GHRH) and somatostatin. Despite that IGF1 is commonly believed to be a mediator of GH function, GH receptors (GHR) are expressed in several tissues (e.g., liver, cartilage cells and growth plates of the long bones), suggesting a direct role of GH [5]. GHRs have also been isolated in the testis. This suggests that GH may play a direct role at this level [6]. However, the role of GH on the testicular function has not been investigated.

Recently, an in-vitro pre-pubertal testis-like organ culture system that is highly responsive to human gonadotropins was developed from neonatal porcine testis [7]. This system has been shown to be capable of preserving human sperm viability for up to seven days [8]. It has been proposed as a new model for experimental studies to understand endocrine issues concerning SCs’ or Leydig cells’ responsiveness to hormone stimuli [7]. Highly-purified FSH (hpFSH) was able to stimulate inhibin B and to inhibit AMH mRNA and protein secretion from SCs in this model [7]. 

Little is known about the action of GH and IGF1 on porcine SCs. These hormones, which are known to support body growth, are mainly secreted early in mammal life, showing multiple tissue-specific roles [9]. At this time, the testis undergoes physiological changes, consisting of proliferation of immature SC, growth and hormone secretion. These changes precede pubertal SC maturation and the achievement of their final number, as well as the beginning of spermatogenesis [1]. On this account, the aim of this study was to evaluate the in-vitro effects of GH and IGF1 on basal and FSH-modulated SC proliferation, AMH and inhibin B expression and secretion.

## 2. Experimental Section

### 2.1. Ethics Statement

This study was carried out in strict compliance with the Guide for the Care and Use of Laboratory Animals of the National Institutes of Health and Perugia University Animal Care. The protocol was approved by the internal Institutional Ethics Committee (Ministry of Health authorization n. 971/2015-PR, 9/14/2015).

### 2.2. Sertoli Cell Isolation, Culture, Characterization, and Function

SCs were obtained from neonatal pre-pubertal Large White pigs (7–15 days of age) and isolated according to established methods [10]. The fibrous capsule was removed. Then, the testes were finely chopped and digested twice enzymatically, with a mixed solution of trypsin and deoxyribonuclease I (DNase I) in Hanks’ balanced salt solution (HBSS; Merck KGaA, Darmstadt, Germany) and collagenase P (Roche Diagnostics S.p.A., Monza, Italy). The tissue pellet was centrifuged, passed through a 500 μm pore stainless steel mesh and then resuspended in glycine to eliminate residual Leydig and peritubular cells [11]. The pellet was then collected and kept in HAM’s F12 medium (Euroclone, Milan, Italy) and added with 0.166 nmol L^−1^ retinoic acid (Sigma-Aldrich, Darmstadt, Germany) and 5 mL per 500 mL insulin–transferrin–selenium (ITS, Becton Dickinson cat. no. 354352; Franklin Lakes, NJ, USA) in 95% air/5% CO_2_ at 37 °C. Cells were cultured for 3 days. Then, the purity and the functional competence of SC monolayers were assessed, as described elsewhere [6]. 

### 2.3. Culture and Treatment

After 3 days of culture, when SC monolayers were confluent, they underwent the following treatments: (1) no treatment; (2) 100 nM highly purified urofollitropin (hpFSH) (Fostimon^®^, IBSA Farmaceutici Srl, Rome, Italy) for 48 h; (3) 100 nM recombinant growth hormone(rGH) (Saizen 8 mg click.easy, Merck Serono S.p.A.) for 48 h; (4) 100 nM recombinant insulin-like growth factor 1 (r-IGF1) (Sigma Life Science) for 48 h; (5) 100 nM hpFSH and 100 nM rGH for 48 h; (6) 100 nM hpFSH and rIGF1 for 48 h.

### 2.4. RT-PCR Analysis

Total RNA was extracted and quantified by reading the optical density at 260 nm. In particular, 2.5 μg of total RNA was subjected to reverse transcription (RT, Thermo Scientific, Waltham, MA, USA) to a final volume of 20 μl. The qPCR was performed using 50 ng of the cDNA prepared by RT and a SYBR Green Master Mix (Stratagene, Amsterdam, The Netherlands—Agilent Technology). This was performed in an Mx3000P cycler (Stratagene) using FAM for detection and ROX as the reference dye. The mRNA level of each sample was normalized against β-actin mRNA and expressed as fold change versus the level in untreated control cells.

The following primers were used for Real-time PCR analysis: AMH, forward primers 5′-GCGAACTTAGCGTGGACCTG-3′, revers primers 5′-CTTGGCAGTTGTTGGCTTGATATG-3′; Inhibin B, forward primers 5′-TGGCTGGAGTGACTGGAT-3′, revers primers 5′-CCGTGTGGAAGGATGAGG-3′; FSHR forward primers 5′-TTTCACAGTCGCCCTCTTTCCC-3′, revers primers 5′-TGAGTATAGCAGCCACAGATGACC-3′; actin, forward primers 5′-ATGGTGGGTATGGGTCAGAA-3′, revers primers 5′-CTTCTCCATGTCGTCCCAGT-3′.

### 2.5. AMH and Inhibin B Secretion Assay

Aliquots of the culture media of treated and untreated SCs were stored at −20 °C for the assessment of AMH (AMH Gen IIELISA, Beckman Coulter; intra-assay CV = 3.89%; inter-assay CV = 5.77%) and inhibin B (Inhibin B Gen II ELISA, Beckman Coulter, Webster, TX, USA; intra-assay CV = 2.81%; inter-assay CV = 4.33%) secretion, as previously described [12].

### 2.6. Cell Number and Proliferation

After reaching 50%–60% confluence, cells were treated with 0.1 µg/mL colcemid (Sigma-Aldrich no. 10295892001) for 3 h in the incubator [13] and washed with phosphate buffer (PBS). For cell proliferation assay, SC were incubated with 1 µM 5(6)-carboxyfluorescein diacetate N-succinmidyl ester (CFSE, catalog no. 21888, Sigma-Aldrich) in PBS for 8 min and washed with HBSS medium three times. Successively, CFSE-labeled SC were cultured at 37 °C, 5% CO_2_ incubator for 48 h following the established protocol of stimulations [hpFSH(100 nM), GH (100 nM), rIGF-1 (100 nM), hpFSH (100 nM) + rGH (100 nM), hpFSH (100 nM) + rIGF1 (100 nM)]. At the end of the stimulation assay, cells were washed with PBS, harvested by tripsinization and then counted using an Automated Cell Counter (Invitrogen, CA, USA) before the flow cytometer analysis [14]. Data acquisition was performed on 20,000 events per tube based on a total (gated alive cells) count of forward and side light scatter at approximately 200–300 events per sec on a BD FAC Sort flow cytometer (BD Biosciences), analyzed using FACS Diva software (BD Biosciences, Franklin Lakes, NJ, USA) and gated on appropriate controls in the different cell populations.

### 2.7. Statistical Analysis

Results are shown as mean ± SD of three independent experiments, each one performed in triplicate. Data were analyzed for statistical significance by one-way ANOVA, followed by a Tukey post hoc test using SPSS 9.0 for Windows (SPSS Inc., Chicago IL, USA). Significance was accepted for *p* values lower than 0.05.

## 3. Results

### 3.1. Cell Number and Proliferation

Compared to the unexposed control, treatment with hpFSH did not significantly change (1.45 ± 0.07% versus 1.5 ± 0.14%) the percentage of dividing cells. Cell proliferation decreased significantly after incubation with rGH (0.85 ± 0.07% versus 1.5 ± 0.14%, *p <* 0.05). In contrast, rIGF1 exposure significantly increased (1.90 ± 0.14% versus 1.5 ± 0.14%, *p <* 0.05) the percentage of divided cells compared to the control. 

**Figure 1:**
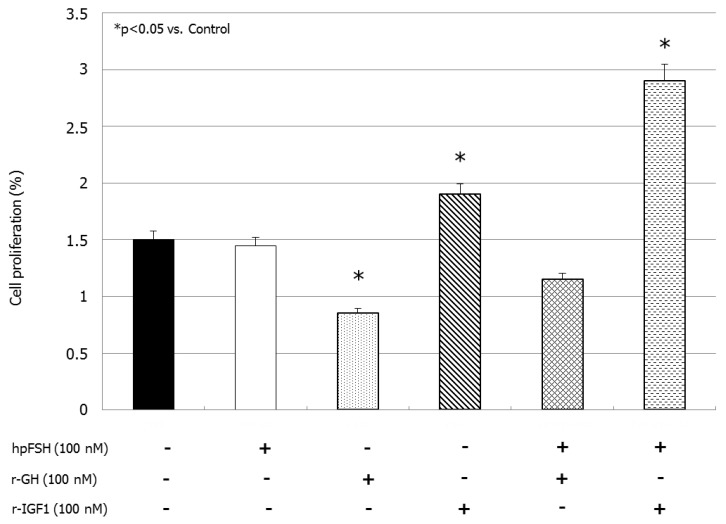
Cell proliferation assay. Data represent the mean ± SD of three independent experiments, each performed in triplicate. (* *p <* 0.05 versus control) (one-way ANOVA). The bars express the percentage of cells with the highest generation number coming from flow cytometric analysis of Sertoli cells stained with CFSE without stimulation after single or combined incubation with hpFSH, rGH or rIGF1.

Treatment with hpFSH and rGH did not modify the percentage of divided cells (1.15 ± 0.21% versus 1.5 ± 0.14%). This suggests that co-incubation with hpFSH may overcome the inhibitory effect of rGH on cell proliferation. Finally, the simultaneous incubation with hpFSH and rIGF1 significantly increased the percentage of divided cells (2.90 ± 0.21% versus 1.5 ± 0.14%, *p <* 0.001) (Figure 1).

### 3.2. FSHR Relative Gene Expression

Treatment with hpFSH or rIGF1 significantly decreased FSHR mRNA levels compared to the control (−41.1% and −46.6%, respectively, *p <* 0.0001). In contrast, rGH significantly increased FSHR mRNA levels (+20.7% versus control, *p <* 0.05). 

Co-incubation with hpFSH and rGH significantly decreased FSHR mRNA levels (−31.6%, *p <* 0.0001), suggesting that the stimulatory effects of rGH were counterbalanced by hpFSH. Finally, co-treatment of cell cultures with hpFSH and rIGF1 significantly decreased FSHR mRNA levels compared to the control (−45%, *p <* 0.0001) (Table 1).

### 3.3. AMH Gene Expression and AMH Secretion 

hpFSH exposure decreased both AMH mRNA levels (−46.1%, *p <* 0.005) and AMH secretion (63.5 ± 2.7 versus 90.1 ± 1.4 µg/cell, *p <* 0.0001) compared to the control. Treatment with rGH reduced AMH secretion (75.5 ± 11.4 versus 90.1 ± 1.4 µg/cell, *p <* 0.05) but did not change the mRNA levels (+0.07%, *p >* 0.1). Incubation with rIGF1 resulted in a significant decrease of the relative gene expression (−60.0%, *p <* 0.0001) levels by the same extent as hpFSH, whereas its inhibitory effect on AMH secretion (44.6 ± 1.4 versus 90.1 ± 1.4 µg/cell, *p <* 0.0001) was stronger than that caused by hpFSH (44.6 ± 1.4 versus 63.5 ± 2.7 µg/cell, *p <* 0.005). 

**Figure 2:**
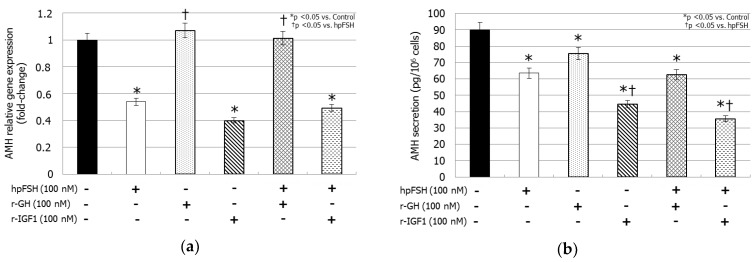
RT-PCR analysis of anti-Müllerian hormone (AMH) gene expression (**a**) and protein secretion (**b**). Data represent the mean ± SD (* *p <* 0.05 versus control and † *p <* 0.05 versus hpFSH treatment) (one-way ANOVA) of three independent experiments, each performed in triplicate.

Co-incubation with hpFSH and rGH did not modify the mRNA levels (+0.01%, *p >* 0.1), but it decreased AMH secretion (62.5 ± 1.8 versus 90.1 ± 1.4 µg/cell, *p <* 0.0001) compared to the control. Finally, compared to the control, treatment with rIGF1 plus hpFSH reduced AMH relative gene expression (−50.7%, *p <* 0.005) by the same extent to that obtained with hpFSH alone, however the suppression of AMH secretion (35.6 ± 5.3 versus 90.1 ± 4.4 µg/cell, *p <* 0.0001) was stronger than hpFSH (35.6 ± 5.3 versus 63.5 ± 2.7 µg/cell, *p <* 0.0001) (Figure 2).

### 3.4. Inhibin B Gene Expression and Inhibin B Secretion 

Treatment with hpFSH increased both inhibin B mRNA levels (+350%, *p <* 0.0001) and that of the secreted protein (25.52 ± 0.52 versus 2.33 ± 0.75 pg/cell, *p <* 0.0001). In contrast, rGH did not modify inhibin B gene expression (−41.5%, *p >* 0.1) and secretion (1.78 ± 0.20 versus 2.33 ± 0.75 pg/cell, *p >* 0.1). Incubation with rIGF1 resulted in increased inhibin B relative gene expression (+318%, *p <* 0.0001) by the same extent compared to that obtained with hpFSH. rIGF1 also increased the levels of the secreted protein compared to the control (28.03 ± 0.19 versus 2.33 ± 0.75 pg/cell, *p <* 0.0001). The raise of inhibin B levels secreted protein was higher compared to hpFSH (28.03 ± 0.19 versus 25.52 ± 0.52 pg/cell, *p <* 0.005).

**Figure 3:**
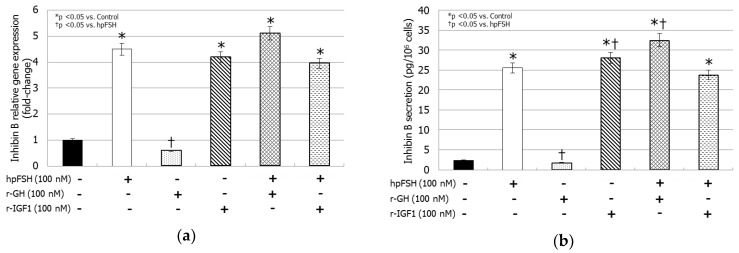
RT-PCR analysis of Inhibin B gene expression (**a**) and protein secretion (**b**). Data represent the mean ± SD (* *p <* 0.05 versus control and † *p <* 0.05 versus hpFSH treatment) (one-way ANOVA) of three independent experiments, each performed in triplicate.

Co-incubation with hpFSH and rGH increased gene expression (+410%, *p <* 0.0001) and protein secretion (32.45 ± 0.75 versus 2.33 ± 0.75 pg/cell, *p <* 0.0001) in comparison to the control. The stimulatory effect on protein secretion was stronger compared to that obtained with hpFSH (32.45 ± 0.75 versus 25.52 ± 0.52 pg/cell, *p <* 0.0001). Finally, the co-incubation with hpFSH and rIGF1 increased both mRNA (+295%, *p <* 0.0001) and protein levels (23.73 ± 0.28 versus 2.33 ± 0.75 pg/cell, *p <* 0.0001) compared to the control, with the same extent as hpFSH alone (Figure 3).

## 4. Discussion

The major objectives of this study were to assess the role of GH and IGF1 on proliferation and hormonal secretion of SCs from neonatal pigs. During infancy and childhood, testis have erroneously been considered as quiescent due to the low activity of the hypothalamic–gonadotropic axis. However, at this time, SCs are the most numerous, actively proliferating and hormone-secreting cells, and the main cellular component of the testicular volume [14,15]. Different from the hypothalamic–gonadotropic axis, the hypothalamic–somatotropic one is active in childhood [9]. This activity has been regarded as having many tissue-specific functions, being that IGF1R is widely spread in the mammal organism [9], including SCs [16]. Therefore, we sought to investigate whether the hypothalamic–somatotropic axis may influence porcine neonatal testicular SCs. The results of this study showed that both GH and IGF1 were capable of influencing porcine SC proliferation and function, but in a slightly different manner. 

Under the experimental conditions used, we found a suppressive effect of GH on SC proliferation. Moreover, it did not affect AMH mRNA but suppressed AMH protein levels. In contrast to FSH, which enhanced inhibin B expression and secretion, GH did not have any significant effect on inhibin B mRNA and protein secretion. The different role of GH and FSH on SC hormone secretion may be further supported by the increased *FSHR* expression in GH-incubated cultures. Furthermore, the FSH-dependent effects on cell proliferation, AMH and inhibin B were not influenced by GH.

To our knowledge, this is the first study investigating the effects of GH on SC proliferation and hormone synthesis. The presence of GH receptors in SCs has already been described [17]. GH has been suggested to play a role in gametogenesis. It was detected in SCs of Japanese eel and its receptors are expressed in germ cells. In a testicular organ culture system, GH stimulation induced germ cell proliferation independently from IGF1 [18]. It was also abundantly detected in secondary spermatocytes and spermatids, myocytes and interstitial cells as well in chicken testis [19]. Furthermore, experimental evidences have shown that the paracrine secretion of GH-releasing hormone (GHRH) from Leydig and germ cells promotes cAMP production in rat SC cultures synergistically with FSH [18]. GHRH paracrine secretion might also induce GH synthesis in SCs, thus in turn stimulating spermatogenesis. Combining these findings with our results, it appears that SC function is influenced by GH. More in detail, GH seems to influence AMH synthesis in immature SCs and to promote spermatogenesis in mature ones. Whether these effects mainly depend on pituitary- or testis-derived GH is not known. Since human pituitary GH is a ~20 kDa protein, its possible function within the testis would be subordinated by the capacity to cross the testicular blood barrier. 

Different from GH, at least at the dosage used in this study, IGF1 enhanced SC proliferation, whereas, surprisingly, FSH alone did not have any effect on this parameter at least at the end of the 48-hour incubation. Similarly to FSH, IGF1 suppressed AMH and increased inhibin B expression and secretion. FSH and IGF1 had similar effects that persisted with the same intensity in co-incubated cultures. Accordingly, *FSHR* was downregulated in cultures incubated with IGF1. In-vitro data indicate a stimulatory effect of IGF1 on germ cell proliferation [16]. Studies in Newt testis [20,21] show a high IGF1 expression in SCs, which is additionally increased after FSH incubation. This brought to hypothesize that FSH-induced spermatogenesis may rely on a paracrine synthesis of IGF1 from SCs [21]. Studies in mutant mice suggest the importance of IGF1 signaling in gonadal male development during embryogenesis [22], as well as in the establishment of a normal-sized SC cohort [4]. Herein, we report the stimulatory effect of IGF1, both alone and in combination with FSH in porcine SCs. Similar results have recently been reported in cultures from pre-pubertal bovine SCs, where the co-incubation with IGF1 and FSH increased cell proliferation and number by ~1.5 fold [23]. Interestingly, and in agreement with such findings, IGF1 serum levels have been found to positively correlate with testicular volume in men [23]. We are not aware of in-vitro studies investigating the effect of IGF1 on AMH and inhibin B secretion from SCs. The similar-to-FSH effects on hormone synthesis are not in contrast with a possible, already suggested IGF1-mediated role on FSH action [16]. The low molecular weight of human IGF1 (~7 kDa) makes it likely that this protein crosses the testicular blood barrier. Hence, a possible pituitary, as well as testis-derived IGF1 role in testicular development and function may be hypothesized. 

## 5. Conclusions

In conclusion, these data indicate that GH and IGF1 are able to influence basal and FSH-modulated SC function. In line with previous evidence [23], we found a stimulatory effect of IGF1, but not of FSH or GH alone, on SC proliferation. Furthermore, both GH, IGF1 and FSH suppressed AMH secretion, whereas IGF1 and FSH, but not GH, stimulated inhibin B synthesis. Wherefore, SC proliferation occurring in childhood may likely be supported by serum IGF1 levels, which are high during this period of life. Since an appropriate number of SCs is needed for spermatogenesis, a lack of IGF1 might compromise SC proliferation and future fertility. The possible relationship between the hypothalamic–somatotropic axis and testis development, AMH and inhibin B serum levels in childhood may, therefore, deserve further investigation. 

## Figures and Tables

**Table 1:** FSHR relative gene expression in stimulated cultures.

Hormones	Fold Change versus Control (mean ± SD)
hpFSH	0.059 ± 0.06 *
r-GH	1.21 ± 0.12 **
r-IGF1	0.53 ± 0.01 **
hpFSH plus r-GH	0.68 ± 0.03 **
hpFSH plus r-IGF1	0.55 ± 0.00 **

**Abbreviations:** hpFSH: highly purified follicle-stimulating hormone; r-GH: recombinant growth hormone; r-IGF1: recombinant insulin-like growth factor 1. * *p <* 0.05; ** *p <* 0.001. The mRNA level of each sample was normalized against β-actin mRNA and expressed as fold change versus the level in untreated control cells.
